# Multiparametric Functional MRI: Non-Invasive Imaging of Inflammation and Edema Formation after Kidney Transplantation in Mice

**DOI:** 10.1371/journal.pone.0162705

**Published:** 2016-09-15

**Authors:** Katja Hueper, Marcel Gutberlet, Jan Hinrich Bräsen, Mi-Sun Jang, Anja Thorenz, Rongjun Chen, Barbara Hertel, Amelie Barrmeyer, Martina Schmidbauer, Martin Meier, Sibylle von Vietinghoff, Abedalrazag Khalifa, Dagmar Hartung, Hermann Haller, Frank Wacker, Song Rong, Faikah Gueler

**Affiliations:** 1 Institute for Diagnostic and Interventional Radiology, Hannover Medical School, Hannover, Germany; 2 Institute for Pathology, Hannover Medical School, Hannover, Germany; 3 Department of Nephrology, Hannover Medical School, Hannover, Germany; 4 Institute for Laboratory Animal Science, Hannover Medical School, Hannover, Germany; 5 The Transplantation Center of the affiliated hospital, Zunyi Medical College, Zunyi, China; 6 The kidney disease centre of the First Affiliated Hospital, Zhejiang University, Hangzhou, China; University Medical Center Utrecht, NETHERLANDS

## Abstract

**Background:**

Kidney transplantation (ktx) in mice is used to learn about rejection and to develop new treatment strategies. Past studies have mainly been based on histological or molecular biological methods. Imaging techniques to monitor allograft pathology have rarely been used.

**Methods:**

Here we investigated mice after isogenic and allogenic ktx over time with functional MRI with diffusion-weighted imaging (DWI) and mapping of T2-relaxation time (T2-mapping) to assess graft inflammation and edema formation. To characterize graft pathology, we used PAS-staining, counted CD3-positive T-lymphocytes, analyzed leukocytes by means flow cytometry.

**Results:**

DWI revealed progressive restriction of diffusion of water molecules in allogenic kidney grafts. This was paralleled by enhanced infiltration of the kidney by inflammatory cells. Changes in tissue diffusion were not seen following isogenic ktx. T2-times in renal cortex were increased after both isogenic and allogenic transplantation, consistent with tissue edema due to ischemic injury following prolonged cold ischemia time of 60 minutes. Lack of T2 increase in the inner stripe of the inner medulla in allogenic kidney grafts matched loss of tubular autofluorescence and may result from rejection-driven reductions in tubular water content due to tubular dysfunction and renal functional impairment.

**Conclusions:**

Functional MRI is a valuable non-invasive technique for monitoring inflammation, tissue edema and tubular function. It permits on to differentiate between acute rejection and ischemic renal injury in a mouse model of ktx.

## Introduction

Magnetic resonance imaging (MRI) allows non-invasive and contrast-free longitudinal examination of renal allografts. Besides imaging of renal morphology, the standard in the clinics, novel functional MRI (fMRI) techniques allow one to assess the structure and function of the transplant[1–5].

After kidney transplantation (ktx) inflammatory processes associated with activation of pro-inflammatory cytokines, cell infiltration and edema formation play an important role in the development of ischemia reperfusion injury (IRI), graft dysfunction, and acute allograft rejection[6–8]. Early diagnosis of graft rejection will be important in patients. Those with the problem should be treated with immunosuppressive agents; those who do not reject their transplants should not.

Mouse models of ktx are frequently used to study mechanisms underlying allograft pathology and to test novel treatment strategies[6, 9]. In these models repetitive fMRI detecting localization and severity of renal inflammation may improve the understanding of disease mechanisms and the effects of therapeutic interventions. In addition, application of fMRI in experimental studies may reduce the number of experimental animals needed by facilitating longitudinal studies in the same animal and pinpointing time points of interest for histological examination.

For the present fMRI study we used diffusion-weighted imaging (DWI) and mapping of T2-relaxation time (T2-mapping) to investigate the severity and course of inflammation and edema formation following ktx in mice. DWI focuses on Brownian motion of water protons within the tissue and is quantified by the apparent diffusion coefficient (ADC)[10]. In tissue with high cellularity, e.g. due to inflammatory cell infiltration, or fibrosis ADC is reduced reflecting restricted diffusion of water protons[11]. This has been shown in mouse models of ischemia-induced acute kidney injury and unilateral ureteral obstruction[12, 13]. In clinical studies, reductions in ADC were seen in renal allografts with impaired renal function[2, 3, 14] and correlated with the extent of graft fibrosis in biopsy specimens[5].

T2-maps of renal tissue display changes in T2 relaxation times in the kidney. These are related to tissue water content[15, 16]. Increased T2-times reflect higher tissue water content driven by capillary leakage and tissue edema. In addition, T2-mapping allows you to image renal compartments such as renal cortex, outer (OSOM) and inner stripe of the outer medulla (ISOM) and inner medulla[12]. Therefore, renal pathology within each compartment and alterations of the physiological gradient of water content from low in the cortex to high in the inner medulla may be determined. In small animal models of IRI induced acute kidney injury, the greatest increases in T2-values have been observed in the OSOM where they are associated with tissue edema[12, 17].

The purpose of this experimental study was to assess the utility of fMRI techniques for non-invasive imaging of inflammation and edema formation due to IRI and acute renal allograft rejection in established mouse models of isogenic and allogenic ktx. We correlated fMRI findings with renal histology and immunohistochemistry the mouse models.

## Materials and Methods

### Animals

This study was carried out in strict accordance with the recommendations in the Guide for the Care and Use of Laboratory Animals of the National Institutes of Health. The protocol was approved by the Committee on the Ethics of Animal Experiments of the Lower Saxony State Office for Consumer Protection and Food Safety (approval number: 33.14-42502-04-14/1569) and was in accordance with the guidelines of Hannover Medical School. Inbred male C57BL/6J^Han-ztm^ (H2^b^) (B6) and female BALB/c J^Han-ztm^ (H2^d^) (BALB/c) mice (12–14 weeks old) weighing 20–26 g were supplied by the Institute of Laboratory Animal Science, Hannover Medical School (Hannover, Germany). Animals were cared for in accordance with institutional guidelines for experimental animals and with the guidelines of the American Physiological Society. Mice were housed in a room with a 14/10 hour light/dark cycle and had free access to food and drinking water. Following ktx, the health and activity of the mice were monitored daily.

### Kidney transplantation in mice

Fully mismatched allogenic ktx was performed with B6 mice as kidney donors and female BALB/c mice as recipients. For isogenic ktx B6 mice served as donors and recipients. A microsurgeon with >15 years of experience performed all surgeries under isoflurane inhalation anaesthesia and butorphanol (1 mg/kg s.c.) for analgesia as described previously[18]. Briefly, the donor kidney was removed with the renal vein, a cuff of the aorta, and the ureter. The recipient’s left kidney was removed and the donor kidney was placed into the left lower abdomen. The vessels of the graft were anastomosed to the recipient’s infrarenal aorta and the inferior vena cava. The ureter was implanted into the bladder. Cold and warm ischemia times were 60 min and 30 min, respectively, in both groups. The right kidney of the recipient was left untouched in order to maintain renal function for the entire the experiment. This allowed recipients with mismatched kidneys to survive the procedure. As a result, longitudinal fMRI measurements could be made, but creatinine and BUN remained unchanged and could not be used to study renal function.

Seventeen isogenic and 15 allogenic ktx were performed and pathophysiological changes were monitored by functional MRI 1 and 6 days after surgery. Nineteen normal B6 mice were studied as well. Mice were sacrificed on day 7. The kidneys were removed and examined. Morphological changes were compared to alterations observed with fMRI.

### Functional MRI for assessment of renal allograft pathophysiology

To assess renal allograft pathology normal mice and animals that had received allogenic and isogenic kidney transplants were examined by fMRI 1 and 6 days postoperatively. Imaging was performed on a 7 tesla small-animal MRI scanner (Bruker, Pharmascan, PHS701, Ettlingen, Germany) and with a circularly polarized volume coil (Bruker T10327 V3) or a 4-channel surface receive coil (Bruker T20027 V3). Anaesthesia was induced with 3% isoflurane and maintained with 1–2% isoflurane during fMRI. Respiration was monitored and kept between 50–60 breaths per minute.

DWI was acquired in coronal orientation using a respiratory-triggered, fat-saturated echo-planar sequence with the following parameters: field of view (FOV) = 35 x 35 mm^2^, slice thickness = 1.2–2 mm, matrix = 128x128—172x172, effective repetition time (TR)/ echo time (TE) = 2000-3000/ 22–35 ms, 7 b-values = 0–800 s/mm^2^. Parameter maps of the apparent diffusion coefficient (ADC) were calculated with MATLAB software (MathWorks, Natick, MA, USA) using a mono-exponential fit as described previously [12]. Mean ADC values were determined for renal cortex and outer medulla on a region of interest (ROI) based analysis using Osirix software (v.6.0.2, Pixmeo, Switzerland). A differentiation of inner (ISOM) and outer stripe of the outer medulla (OSOM) was not possible on ADC maps.

For T2-mapping a fat-saturated, respiratory-triggered multi-echo turbo-spin-echo sequence was acquired in coronal orientation: FOV = 35 x 35 mm^2^, slice thickness = 2 mm, matrix = 256x256, TR = 2000–3000, 7 TE = 11–77 ms. Maps of T2-relaxation time were calculated with MATLAB software (MathWorks, Natick, MA, USA)[12, 19] and mean values for renal cortex, outer (OSOM) and the inner stripe of the outer medulla (ISOM) were determined.

MRI data was analysed by two independent readers who were blinded to the results of the other reader and the animal group identity.

### Histology and immunohistochemistry for assessment of inflammation

Seven days after ktx (i.e. one day after the second MRI examination), animals were sacrificed and their kidneys were removed. Under general anesthesia, the mice were perfused with ice-cold phosphate-buffered saline (PBS) via the left ventricle, causing circulatory arrest. Kidneys were harvested and dissected into two parts. One part was fixed in formalin, the other was stored in PBS and processed for flow cytometric analysis.

Formalin fixed tissue was embedded in paraffin and 2 μm sections were cut for PAS staining to assess morphology and to score for signs of rejection according to 2013 Banff criteria[20] by a nephropathologist blinded to the animal group assignment. In addition, immunohistochemistry for CD3 (rabbit anti human 1:250, DAKO) was performed to identify T-lymphocytes. Scoring was done semi-quantitatively at 200-fold magnification: 0 = no CD3 positive infiltrates, 1 = mild infiltration with <5 cells per view field (VF), 2 = moderated infiltration 6–15 cells/ VF, 3 = severe infiltration 16–25 cells/VF, 4 = very severe infiltration ≥ 26 cells/ VF. General inflammation was scored in PAS stained tissue: 0 = no inflammation, 1 = mild inflammation of <10% of the tubulointerstitial area, 2 = moderated inflammation 11–25%, 3 = severe inflammation 26–50%, 4 = very severe infiltration >51%.

### Cell preparation and staining for flow cytometry

Kidney tissue was digested with a cocktail of 250 U/ml collagenase XI, 450 U/ml collagenase I, 120 U/ml DNase I, and 120 U/ml hyaluronidase (Sigma-Aldrich, and Merck KGaA, Darmstadt, Germany). Single cell suspensions were obtained by the use of a 70 μM cell strainer. The following labeled anti-murine antibodies were used: CD45 (30-F11), CD19 (6D5), TCR-β (H37-597). Antibodies were purchased from BD Biosciences (Heidelberg, Germany) or BioLegend (San Diego, CA). Near infrared LIVE/DEAD Fixable Dead Cell Stain Kit (Invitrogen, Carlsbad, CA) was used according to the manufacturer’s instructions. Flow cytometry analysis was performed on a BD Biosciences FACS Canto. Data were analyzed using DIVA software (BD Biosciences). Gating was set for live dead (LD) negative, CD45 positive cells.

### Statistical analysis

Statistical analysis was performed with GraphPad Prism 6.0 (GraphPad Software, Inc., La Jolla, CA, USA) and SPSS version 23 (IBM Corporation, USA). As MRI parameters were not normally distributed according to the Kolmogorov-Smirnov test, we used the non-parametric Kruskal-Wallist test to compare functional MRI parameters and renal histology between groups of allogenic and isogenic ktx and the control group. Adjustment for multiple comparisons was performed with the Dunn’s test. All values are given as mean and standard deviation (SD). Interobserver variability of MRI parameters in ktx mice was tested by the intraclass correlation coefficient (ICC). The correlation between MRI parameters and inflammation scores at histology was determined with Spearman’s test. In addition, receiver operating curve (ROC) analysis was performed to test the diagnostic accuracy of functional MRI parameters to discriminate allografts with acute rejection from isografts without rejection. The area under the curve (AUC) as well as sensitivities and specificities at the Youden selected threshold were determined.

P-values <0.05 were accepted as significant.

## Results

Animals with surgical complications (hind limb paralysis due to thrombus formation at the arterial anastomosis, severe ureteral dilation) and obvious distress (reduced activity, impaired food intake) were sacrificed. In addition, animals with complete infarction of the graft were excluded. Consequently, 13 and 9 isogenic ktx animals could be studied on days 1 and 6, respectively. Twelve and 11 allogenic ktx mice were available for analysis on day 1 and day 6, respecitvely.

### Histology and immunohistochemistry in transplant kidneys

#### Acute rejection and acute tubular injury following kidney transplantation

**Fig 1:**
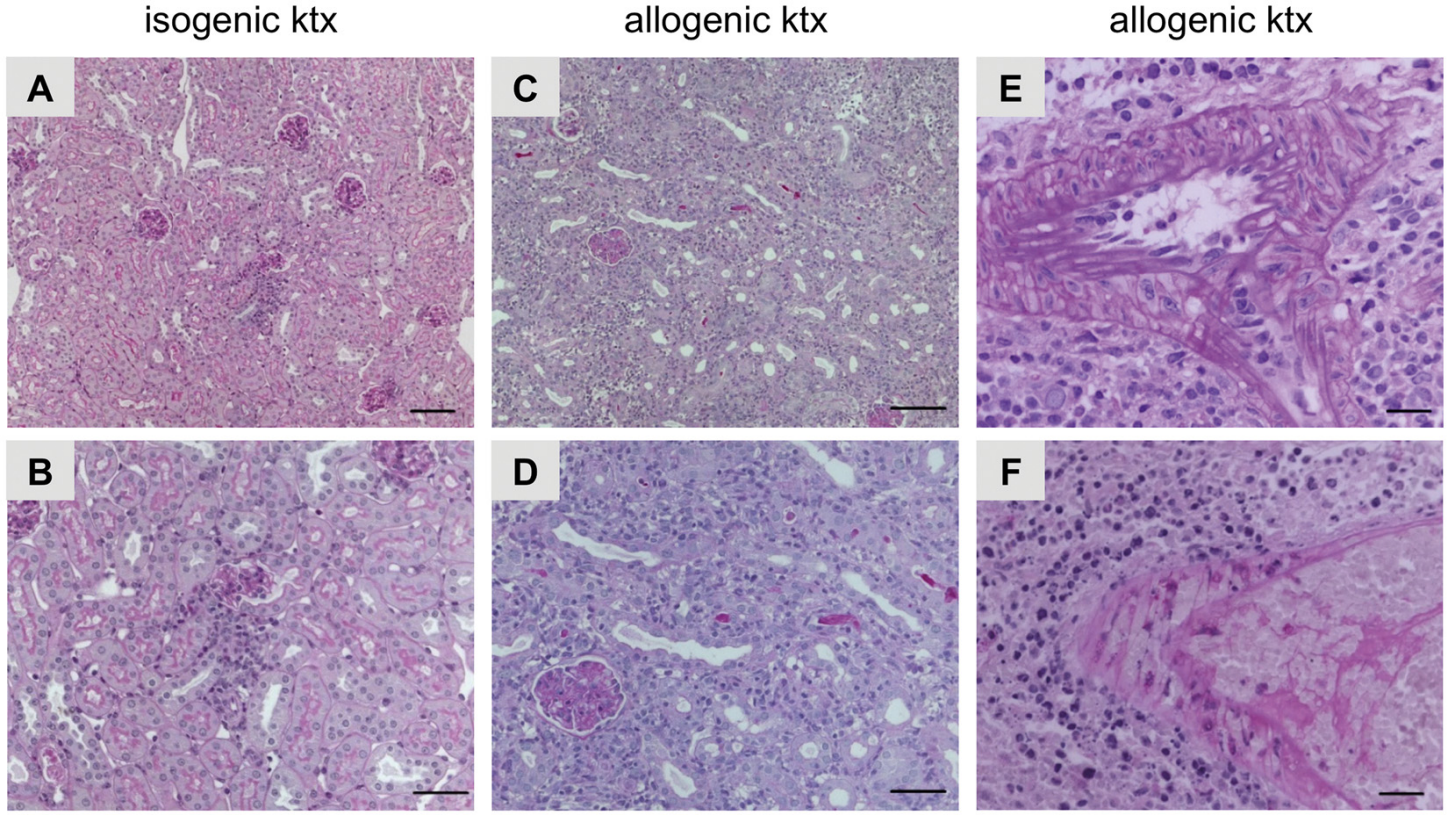
Histopathology in isogenic and allogenic kidney grafts. PAS stainings illustrating histopathology of mouse isografts (A, B) and allografts (C-F). Isografts showed almost normal renal morphology with some focal interstitial inflammatory infiltrates (A and B). Mouse allografts revealed severe rejection (C and D). In addition, allografts with arterial endothelialitis (Banff IIA, E) and transmural arteriitis (Banff III, F) are shown. Bars represent 20 μm in A and B, 50 μm in B and D and 100 μm in E and F.

Allogenic ktx caused T-cell mediated rejection (Banff IA, IB, IIA, IIB, III), while isogenic ktx was associated with mild inflammation without the characteristics of rejection. In addition, in allogenic kidney grafts arterial endothelialitis (Banff IIA) and in some cases transmural arteriitis (Banff III) was observed (Fig 1).

#### Inflammation following kidney transplantation

**Fig 2:**
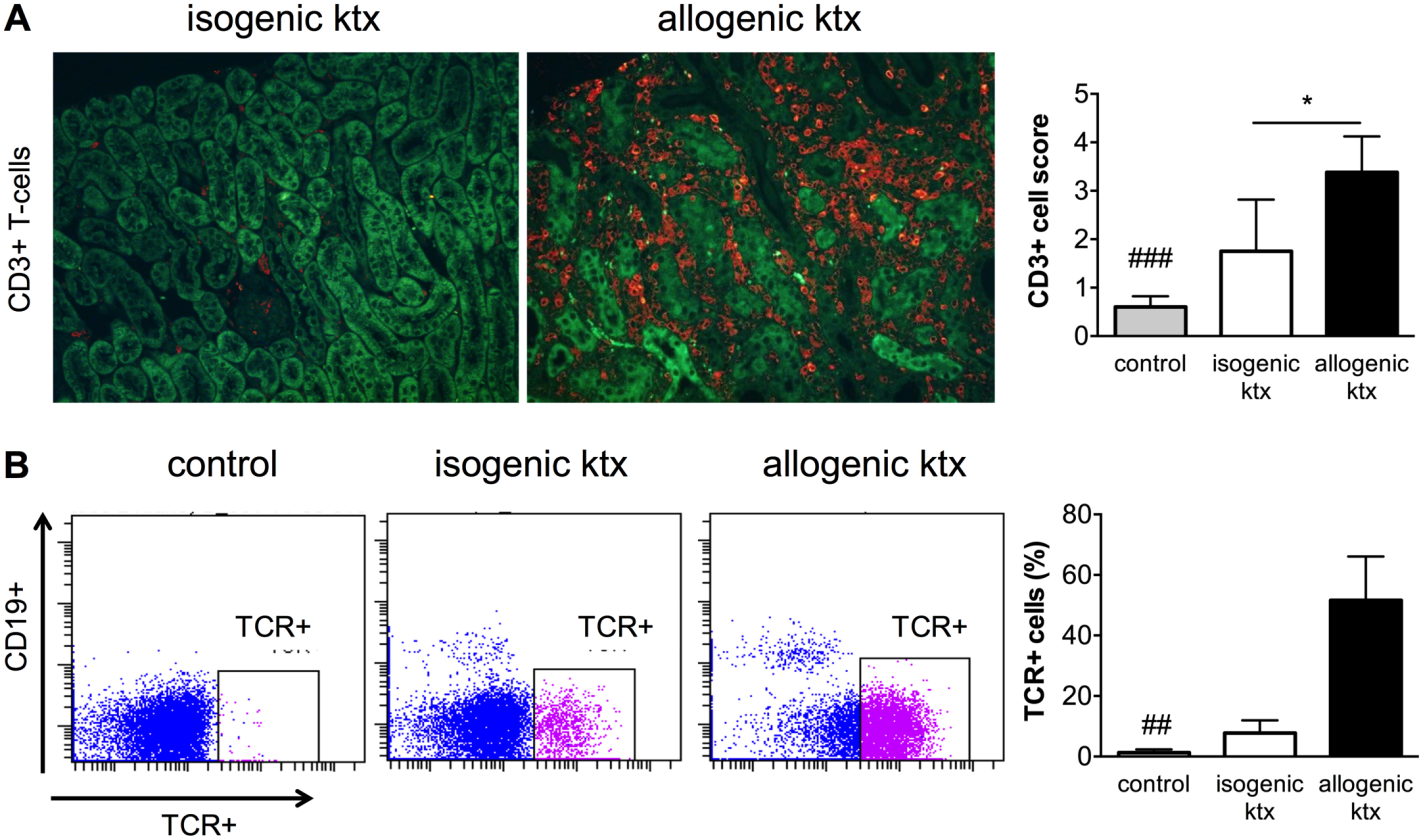
Characterization of infiltrating leukocyte subtypes. Immunohistochemistry with staining for CD3-positive cells showed mild baseline CD3-positive T-lymphocyte expression isogenic kidney grafts. Allogenic kidney grafts had dense infiltrates of CD3-positive lymphocytes (red) at day 7 after transplantation (magnificatin 200-fold). The tubular autofluorescence is overlaid in green for anatomical orientation. Flow cytometric analysis confirmed that in allogenic ktx βTCR-positive (TCR+) cells were the most abundant leukocyte subset, while in isogenic ktx slightly enhanced TCR+ cell infiltration was present (gating examples among live, CD11b+ cells). More details on the cell sorting process are provided in S1 Fig. *p<0.05, ## p<0.01 and ### p<0.001 in comparison to allogenic group.

Ongoing inflammation with infiltration of T-lymphocytes is a hallmark of allogenic rejection. We characterized the leukocyte subtypes by immunohistochemistry as well as flow cytometric analysis. CD3-positive T-lymphocytes were the most abundant cell type 7 days after allogenic ktx, whereas in kidney grafts after isogenic ktx CD3-positive cell numbers were only slightly increased compared to those in control kidneys (Fig 2A). Flow cytometric analysis confirmed this; αβT-cell receptor positive cells (TCR+) contributed up to 50% of the infiltrating cells in allogenic kidney grafts (Fig 2B). CD19-positive B-lymphocyte counts were negligible in both groups at that time point. The comparison with the contralateral native kidney of the recipients showed that TCR+ cells were also slightly more common in the native kidney of recipients after allogenic ktx but not after isogenic ktx (data not shown).

### Functional MRI in transplant kidneys

#### Diffusion-weighted imaging—evaluation of inflammation and cell infiltration

**Fig 3:**
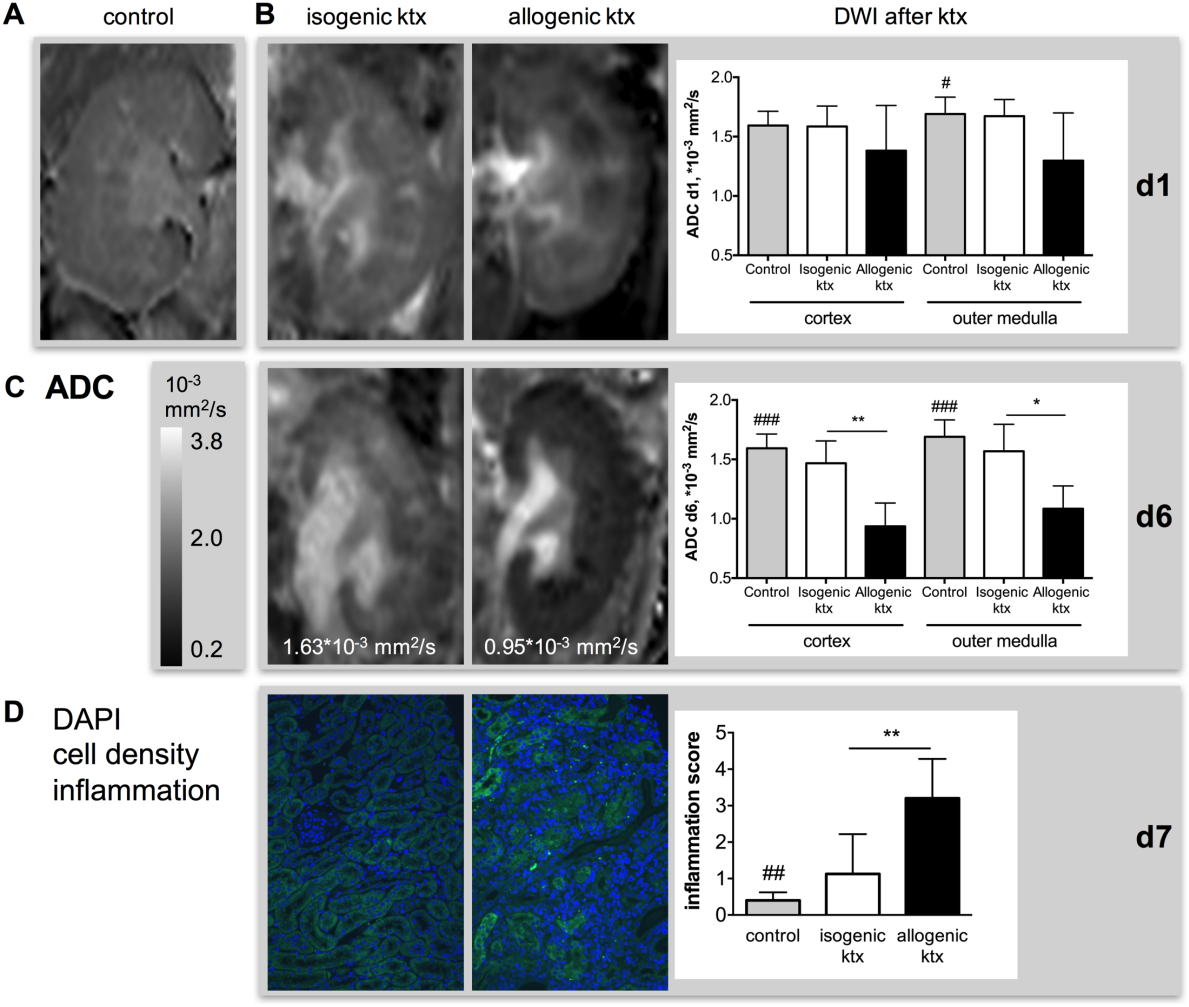
Inflammation and cell infiltration in isogenic and allogenic kidney grafts measured by DWI and histology. Representative ADC maps of a control kidney (A, first column) and isogenic (second column) and allogenic kidney grafts (third column) at day 1 (B) and day 6 (C) are shown after adjustment for multiple comparisons. Mean ADC values in cortex and outer medulla as well as significant group differences are indicated. Corresponding to ADC reduction in allogenic kidney grafts histology revealed elevated tubulo-interstitial inflammation. This correlated with enhanced DAPI positive nuclei visualized in blue (D). Representative DAPI stains, overlaid with tubular autofluorescence for anatomical orientation, of an isograft (second column) and an allograft (third column) are shown. Inflammation was scored semi-quantitatively in PAS stained sections (magnificatin 200 fold). *p<0.05, **p<0.01, ## p<0.01 and ### p<0.001 compared to allogenic kidney grafts.

Interobserver agreement of ADC values in cortex and outer medulla was excellent with ICC of 0.95 and 0.96, respectively. ADC values, representing tissue diffusivity, were significantly reduced after allogenic ktx at day 1 in the renal outer medulla compared to control and isogenic kidneys (allogenic ktx 1.30±0.40*10^-3^ mm^2^/s vs. control 1.69±0.14*10^-3^ mm^2^/s; Fig 3A+3B & Table 1). Six days after ktx, ADC in allogenic kidney grafts was reduced compared to control and isogenic kidneys in renal cortex (allogenic ktx 0.94±0.20*10^-3^ mm^2^/s vs. control 1.59±0.12*10^-^ mm^2^/s vs. isogenic ktx 1.47±0.19*10^-3^ mm^2^/s; p<0.001 and p<0.001) and outer medulla (allogenic ktx 1.08±0.19*10^-3^ mm^2^/s vs. control 1.69±0.14*10^-3^ mm^2^/s vs. isogenic ktx 1.57±0.23*10^-3^ mm^2^/s; p<0.001 and p<0.001; Fig 3C). ADC in isogenic kidney grafts and control kidneys was not significantly different. Corresponding to ADC reduction in allogenic kidney grafts, histology revealed elevated tubulo-interstitial inflammation (inflammation score allogenic ktx 3.2±1.1 vs. control 0.4±0.2 vs. isogenic ktx 1.1±1.1; Fig 3D) and enhanced DAPI positive nuclei visualized in blue (Fig 3D). There was a trend towards negative correlations between ADC in cortex and outer medulla and inflammation scores at PAS histology (r = -0.45, p = 0.11 and r = -0.46, p = 0.10, respectively).

**Table 1:** Functional MRI parameters after isogenic and allogenic ktx and in control animals.

	Control	Isogenic ktx		Allogenic ktx	
		d1	d6	d1	d6
ADC cortex, *10^-3^ mm^2^/s	1.593 ± 0.120	1.586 ± 0.171	1.468 ± 0.189	1.381 ± 0.383	0.936 ± 0.197***^,^^##^
ADC medulla, *10^-3^ mm^2^/s	1.690 ± 0.142	1.673 ± 0.139	1.567 ± 0.229	1.297 ± 0.402*	1.083 ± 0.192***^,^^#^
T2 cortex, ms	38.3 ± 2.0	50.6 ± 4.4***	49.2 ± 7.4***	47.3 ±4.7***	46.0 ± 3.1***
T2 OSOM, ms	36.2 ± 2.1	51.8 ± 6.0***	49.0 ± 8.5***	50.9 ± 6.9***	48.7 ± 5.6***
T2 ISOM, ms	47.0 ± 5.1	62.5 ± 9.1***	58.4 ± 12.0*	51.9 ± 10.4^#^	46.7 ± 7.7^#^
T2 difference, ms	8.7 ± 3.4	11.9 ± 6.2	9.1 ± 5.3	4.6 ± 9.3^#^	0.6 ± 6.7**^,^^#^

Significant differences compared to control kidney after adjustment for multiple comparisons with the Dunn’s test are indicated with

*p<0.05,

**p<0.01,

***p<0.001.

Significant differences of allografts compared to isografts are indicated with

^#^p<0.05,

^##^p<0.01.

MRI data of each individual animal is provided in S1 Table. T2 difference represents the difference between T2-relaxation time in inner stripe of the outer medulla (ISOM) and the renal cortex.

In a subgroup of control, isogenic and allogenic ktx animals intravoxel incoherent motion (IVIM) analysis was performed to investigate the contribution of perfusion and pure diffusion to ADC changes (S1 Text). Pure diffusion (ADCd) was significantly reduced in cortex and medulla of allogenic kidney grafts when compared to control kidneys and isogenic kidney grafts (p<0.05, S2 Fig). For the perfusion fraction (Fp) a trend towards lower values was observed in both isogenic and allogenic kidney grafts. In the same subgroup, DTI showed reduced fractional anisotropy (FA) in the outer medulla in allografts with acute rejection compared to control kidneys and isogenic grafts (S1 Text and S1 Fig).

#### T2-mapping–evaluation of inflammation and tissue edema

**Fig 4:**
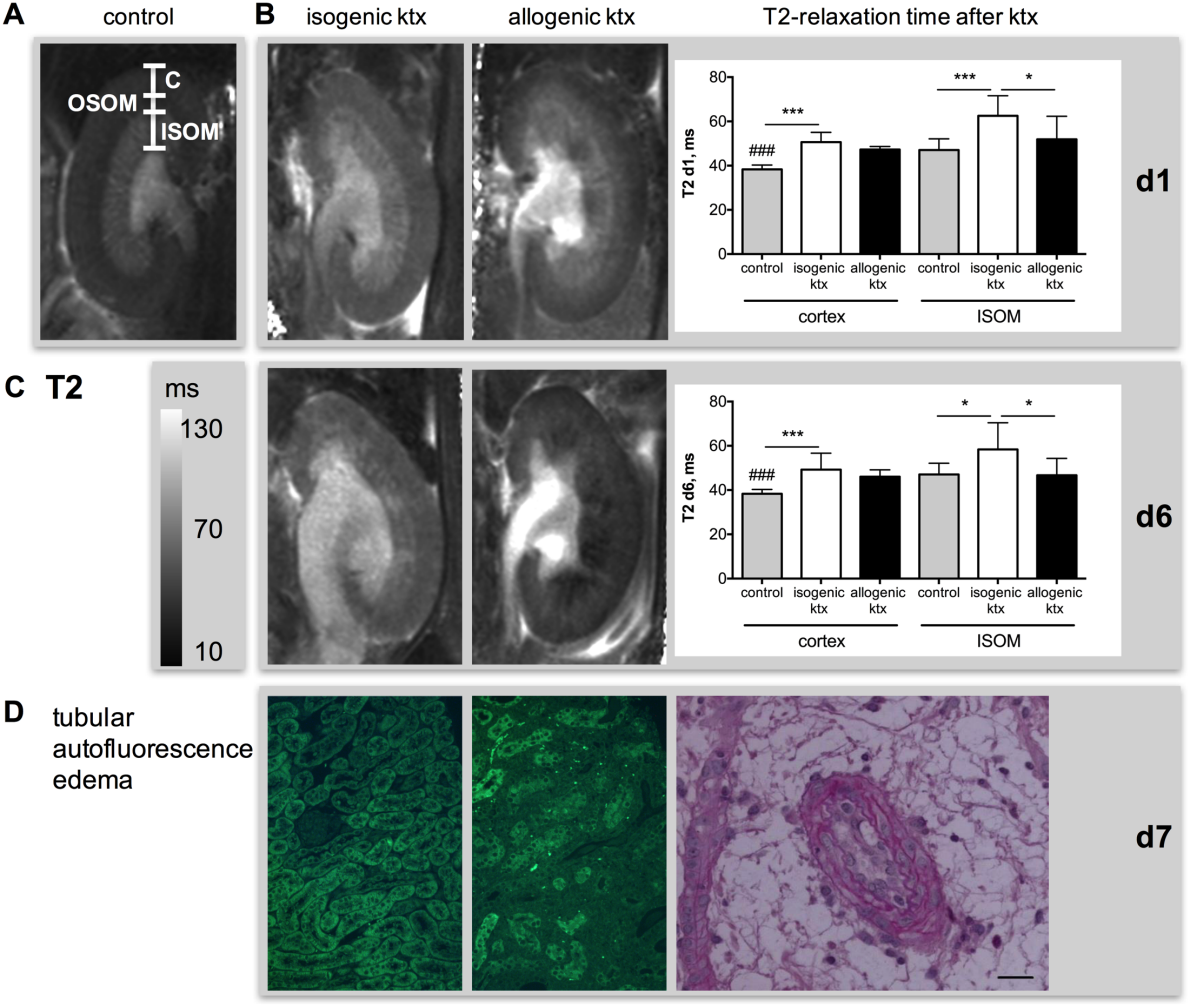
Inflammation and tissue edema in isogenic and allogenic kidney grafts measured by T2-mapping and histology. T2 maps of a control kidney (A, first column) and isogenic (second column) and allogenic kidney grafts (third column) at day 1 (B) and day 6 (C) are shown. Differentiation of renal compartments such as cortex (C), outer (OSOM) and inner stripe of the outer medulla (ISOM) is visualized in panel A. Note the loss of T2-difference between cortex and ISOM in allogenic grafts at d6 after ktx. This corresponds with the loss of tubular autofluorescence (green), which was associated with rejection and loss of tubular function in allogenic ktx (D). PAS stain shows severe endothelialitis and concomitant surrounding edema in allogenic graft (D). Bar represents 50 μm. *p<0.05, **p<0.01, ***p<0.001, ### p<0.001 compared to allogenic kidney grafts.

Interobserver agreement of T2 values in cortex, OSOM and ISOM was excellent with ICC of 0.94, 0.96 and 0.96, respectively. T2-relaxation times increased after ktx in isogenic and allogenic groups in renal cortex and OSOM when compared to control animals indicating tissue edema (Fig 4 & Table 1). In the renal cortex, T2 was increased at day 1 after ktx from 38.3±2.0 ms in control kidneys to 50.6±4.4 ms in the isogenic group (p<0.001) and to 47.3±4.7 ms in the allogenic group (p<0.001). This T2-increase remained stable until day 6. In the isogenic ktx group, T2-relaxation time was also significantly increased in the ISOM, so that the physiological difference between low T2-times in cortex and OSOM and high T2-times in the ISOM was preserved. In the allogenic group, T2-times in the ISOM were not different from those in normal kidneys and were significantly lower than those in isogenic kidney grafts at d1 (51.9±10.4 ms vs. 62.5±9.1 ms, p<0.01) and d6 (46.7±7.7 ms vs. 58.4±12.0 ms, p<0.01). Consequently, the physiological difference between cortex/OSOM and ISOM was abrogated after allogenic ktx (Fig 4C). Histologically, tissue edema was detectable after ktx. In addition, allogenic ktx resulted in loss of tubular autofluorescence indicating acute tubular injury with loss of tubular function (Fig 4D). T2-relaxation time of the ISOM and the T2-difference negatively correlated with inflammation scores at histology (r = -0.64, p<0.05 and r = -0.79, p<0.001).

### Diagnostic accuracy of functional MRI parameters to discriminate allografts with acute rejection from isografts without rejection

**Fig 5:**
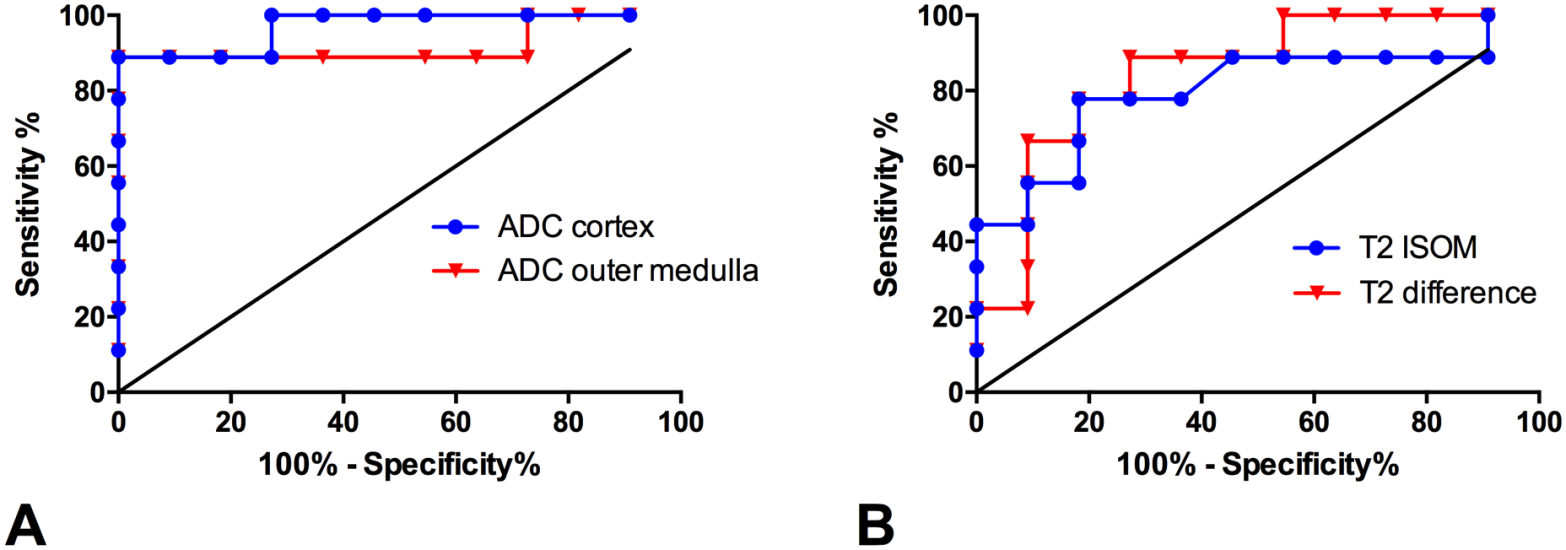
Diagnostic accuracy of functional MRI parameters to discriminate allografts with acute rejection from isografts without rejection. ROC curve analysis shows diagnostic accuracies of ADC values (A) and T2 relaxation times (B) to discriminate allografts with acute rejection from isografts without rejection. Area under the curve, Youden selected thresholds as well as sensitivities and specificities are given in Table 2.

Receiver operating curve (ROC) analysis revealed best diagnostic accuracy to discriminate allografts with acute rejection from isografts without rejection for ADC in the renal cortex at day 6 after ktx with and area under the curve (AUC) of 0.97. At a Youden selected threshold of below 1.315*10^-3^ mm^2^/s sensitivity and specificity were 89% and 100%, respectively (Youden index at threshold 89%, Table 2). Diagnostic accuracy of ADC in the outer medulla was nearly equivalent at a cut-off value of below 1.423*10^-3^ mm^2^/s (Fig 5A). T2 relaxation times were less accurate to discriminate allografts from isografts. Best accuracy was achieved by using the T2-difference between inner stripe of the outer medulla and renal cortex at a threshold below 8.7 ms with a sensitivity of 67% and a specificity of 99% (AUC = 0.85, Youden index at threshold = 66%, Fig 5B).

**Table 2:** Diagnostic accuracy of functional MRI parameters to discriminate allografts with acute rejection from isografts without rejection.

Diagnostic parameter	AUC [95% CI]	Youden selected threshold	Sensitivity at threshold	Specificity at threshold	Youden index at threshold
ADC cortex	0.97 [0.90;1]	1.315*10^-3^ mm^2^/s	89%	100%	89%
ADC outer medulla	0.92 [0.77;1]	1.423*10^-3^ mm^2^/s	89%	100%	89%
T2 cortex	0.64 [0.35;0.92]	47.2 ms	56%	99%	55%
T2 OSOM	0.52 [023;0.80]	55.9 ms	33%	99%	32%
T2 ISOM	0.80 [0.59;1]	52.4 ms	60%	78%	82%
T2 difference	0.85 [0.67;1]	8.7 ms	67%	99%	66%

The diagnostic accuracies to discriminate allografts with acute rejection from isografts without rejection by functional MRI at day six after transplantation are given

ADC = apparent diffusion coefficient, AUC = area under the curve, CI = confidence interval, ISOM = inner stripe of the outer medulla, OSOM = outer stripe of the outer medulla.

## Discussion

This study shows that fMRI is valuable for non-invasive, longitudinal evaluation of inflammation and tissue edema following ktx in mice. Functional MRI enabled clear differentiation of allogenic kidney grafts with acute rejection from isogenic kidney grafts. Progressive ADC reduction in allogenic compared to isogenic grafts and normal kidneys paralleled cellular infiltration, predominantly with T-lymphocytes, and tissue inflammation corresponding to acute rejection histologically. T2-increase, indicating tissue edema, was present in both transplantation groups, most likely due to acute kidney injury of the graft following prolonged cold ischemia time. However, only in the allogenic group the T2-difference of renal compartments—representing the physiological differences of tissue water content—was abrogated. This was paralleled by a loss of tubular autofluorescence and tubular water content presumably as a result of impairment of tubular function due to allograft rejection.

For our experiments, a well-characterized ktx mouse model with 60 min cold and 30 min warm ischemia time was used [6, 7, 9, 18]. The prolonged 60 min cold ischemia time was a trigger for delayed graft function followed by acute T cell mediated rejection (TCMR), so that all animals after allogenic ktx in our study developed interstitial inflammation, tubulitis or endothelialitis according to Banff criteria (IA, IB, IIA, IIB, III). Acute TCMR was characterized in PAS stained tissue sections and infiltration of CD3-positive T-lymphocytes was assessed by immunohistochemistry. Flow cytometric analysis confirmed that TCR+ cells were the most abundant leukocyte subtype one week after ktx in this model. Isogenic ktx with prolonged cold ischemia time of 60 min was associated with mild inflammation, most likely caused by IRI. These changes are comparable to those seen in delayed graft function (DGF) in patients early after kidney transplantation and are a risk factor for acute rejection and impaired long-term allograft survival [21, 22]. Renal function impairment in the mouse model we used could not be assessed by s-creatinine elevation, as the recipient’s right native kidney was not removed. In previous studies, we have shown that removal of both native kidneys in the allogenic model resulted in a 4-6-fold increase in s-creatinine within six days of surgery and normal renal function after isogenic ktx [7, 9].

The obvious and most important drawback of histological analysis in experimental studies of renal transplantation is that animals have to be sacrificed to obtain tissue. Longitudinal evaluation is impossible. Therefore, by providing detailed morphological and functional information and permitting repeated studies of the same animal, fMRI should help us understand mechanisms of allograft rejection and study new therapeutic interventions in ktx mouse models. It also helps to identify relevant time points for histological analysis when these have to be done, reducing the number of animals one has to study. Furthermore, MRI allows us to examine the entire kidney graft and rule out confounding pathologies such as hydronephrosis or infarction of the graft. Focal pathologies are also clearly detectable with fMRI, while they might be missed on histological analysis and in particular in biopsies due to sampling errors. In this study, animals with confounding pathologies were excluded from analysis and focal changes were not included in the quantitative fMRI analysis.

The focus of our study was to evaluate the combination of fMRI techniques DWI and T2-mapping that provide biomarkers for allograft inflammation and to characterize changes of these biomarkers during the development of acute allograft rejection and IRI in comparison to histopathology. This is different from our previous study evaluating a multiparametric MRI protocol including four fMRI techniques to differentiate acute rejection from ischemic injury three weeks after ktx in mice when advanced histopathological changes were present [23].

ADC measured by DWI was reduced on day 1 after allogenic ktx in the outer medulla and ADC reduction proceeded to day 6 and involved the entire kidney. This ADC reduction can be explained by increased overall cellularity visualized on DAPI stains, T-cell infiltration and inflammation histologically in allogenic compared to isogenic kidney grafts and normal kidneys. The association of ADC reduction with these pathological conditions has been shown in different mouse models [12, 13, 23] and in a rat model of ktx [24]. Although histology and immunohistochemistry evidenced mild inflammation and cell infiltration in isografts compared to normal kidneys, ADC values in this group were slightly lower but not significantly different from normal kidneys at day 6 after ktx. This indicates that cell density has to increase above a certain level to be detectable by DWI. ROC curve analysis revealed that ADC reduction in cortex and outer medulla below 1.315*10^-3^ mm^2^/s or 1.423*10^-3^ mm^2^/s, respectively, predicted acute allograft rejection with high sensitivity and specificity of 89% and 100%, respectively. With IVIM in a subgroup of animals we could separate the influence of perfusion and pure diffusion on ADC values. Results show that pure diffusion (ADCd) is reduced in grafts with acute rejection compared to controls and isogenic grafts without rejection, while the perfusion fraction is slightly lower in both transplantation groups. Additional DTI showed reduced diffusion anisotropy in the renal medulla of allogenic kidney grafts, potentially due to changes in renal architecture and tubular damage. However, DWI is not specific for a certain cell type or a specific histological feature but provides a composite marker of all changes that contribute to a restriction of tissue diffusivity such as cell infiltration, cell swelling due to capillary leakage and edema, collagen deposition, and infarction. Therefore, fMRI cannot replace renal histology or detailed cellular and molecular analyses, but it can give an overview of the time course and distribution of inflammatory changes in donor kidneys, and guide histological and molecular studies.

Mapping of T2-relaxation times (T2-mapping) allows excellent discrimination of renal compartments as their tissue water content differs substantially [15, 16]. Cortex and OSOM have low water content, while ISOM and inner medulla have high water content due to the large volume of tubules and collecting ducts. Thus, a compartment-based analysis is important to detect pathologies. In contrast to DWI, T2-mapping clearly detected the ischemic injury in isografts on 1 and 6 days after ktx. However, T2-mapping was less accurate to discriminate between allografts with acute rejection and isografts without rejection compared to DWI (Fig 5). Ischemia was associated with a significant increase in T2, in all renal compartments compared to normal kidneys. We speculate that this is driven by capillary leakage and caused by prolonged cold ischemia time of 60 min (Fig 4D). Similarly, we showed in a model of unilateral IRI that T2-increase of cortex and OSOM is related to ischemia time and peaked seven days after injury [12]. In allogenic kidney grafts, T2-changes are more complex with an increase of T2-time in cortex and medulla and comparable T2-values in the ISOM compared to normal kidneys but significantly decrease compared to isogenic grafts. This results in a loss of the physiological cortico-medullary difference of T2-relaxation time. The cortico-medullary T2-difference inversely correlated with inflammation scores at histology. Increase of T2 in cortex and OSOM parallels changes in isogenic kidney grafts and may therefore be interpreted as tissue edema. Lack of T2-increase in the ISOM indicates that, despite of edema formation, the water content remains stable. A possible explanation is that the water content within tubules and collecting ducts in grafts with acute rejection is lower compared to isogenic grafts due to tubular dysfunction and reduced kidney function. This hypothesis is supported by the loss of tubular autofluorescence in the medulla found at histology. The normal kidney has a large amount of autofluorescence, particularly in the proximal tubule, because of the existence of a number of endogenous fluorophores [25]. The combined measurement of T2-times in different renal compartments enables to discriminate pathologies such as acute rejection with functional impairment and tubular dysfunction and IRI with tissue edema but normal tubular function.

The results obtained in this experimental analysis are translatable into clinical practice [3, 26–28]. The direct correlation of imaging parameters with allograft histology should help us interpret fMRI results in patients with renal allograft pathologies. Nonetheless, one has to consider that renal anatomy and physiology in mice is different from humans and that fMRI parameters at high field strength of 7 T may differ from those at clinical scanners with 1.5–3 T. In particular, fMRI may be useful to identify inflammatory changes of the renal allograft in patients with inconclusive or discrepant biopsy results as well as to guide histopathological sampling and to avoid sampling errors in case of focal allograft changes. Furthermore, repetitive fMRI due to its non-invasive nature is applicable for monitoring of inflammation and tissue edema during the course of disease and treatment.

A limitation of the fMRI techniques used in this study is that they are not specific for certain histopathological changes. However, the combination of DWI and T2-mapping with longitudinal examinations provides an excellent overview of the pathophysiological processes that occur within different compartments of the kidney during acute rejection and IRI and may be useful to guide further detailed histological analysis. Furthermore, we obtained histology only at day 7 after ktx, so that no correlation with fMRI parameters is available on day 1. This is due to the fact that we sought to study longitudinal changes within the same animal and therefore did not plan to sacrifice animals before reaching the endpoint of the experimental study.

In conclusion, our study shows that fMRI offers additional diagnostic tools for longitudinal evaluation of inflammation as a hallmark of renal allograft pathology. DWI provides information about tissue cellularity and correlates with T-cell infiltration and inflammation due to acute rejection observed histologically. T2-mapping, by assessing tissue water content, offers complementary insights into inflammatory processes by determining the degree and localization of tissue edema and indicating tubular integrity and function. The combination of these fMRI techniques is valuable to study the course and the mechanisms of graft inflammation as an important factor during renal allograft rejection and IRI.

## Supporting Information

S1 Fig Details on the flow cytometric analysis. After kidney digestion and washing, cells were gated by scatter properties and live dead stain. CD11b+ myeloid cells, CD19+ B lymphocytes and TCR+ cells were analyzed as % of all leukocytes. T-cell receptor is expressed on both T-cell subtypes CD4 and CD8+ cells. Since by flow cytometric analysis both cell types are detected together the immunohistochemistry marker chosen was also CD3, which is expressed on all T-lymphocytes.
(TIFF)

S2 Fig IVIM analysis and DTI in isogenic and allogenic kidney grafts. Depicted are mean ± SD of IVIM parameters ADCd (pure diffusion, A & B) and Fp (perfusion fraction, C & D) in renal cortex and renal medulla. In addition, FA is shown (fractional anisotropy, E & F). Significant differences are indicated *p<0.05, **p<0.01. Differences between control kidneys and allogenic kidney grafts are indicated with # p<0.05, ## p<0.01.
(TIFF)

S1 Table MRI data per animal. Listed are the measured MRI data in each animal on day 1 and day 6 after transplantation as well as in control animals.
(XLSX)

S1 Text IVIM analysis and DTI. The methods of IVIM and DTI are explained in Supporting File 1.
(DOCX)
