# Supplementary figures and images for: Multiparametric Functional MRI: Non-Invasive Imaging of Inflammation and Edema Formation after Kidney Transplantation in Mice
Source: PLoS One. 2016 Sep 15;11(9):e0162705. doi: 10.1371/journal.pone.0162705 (PMC5025122; doi:10.1371/journal.pone.0162705)

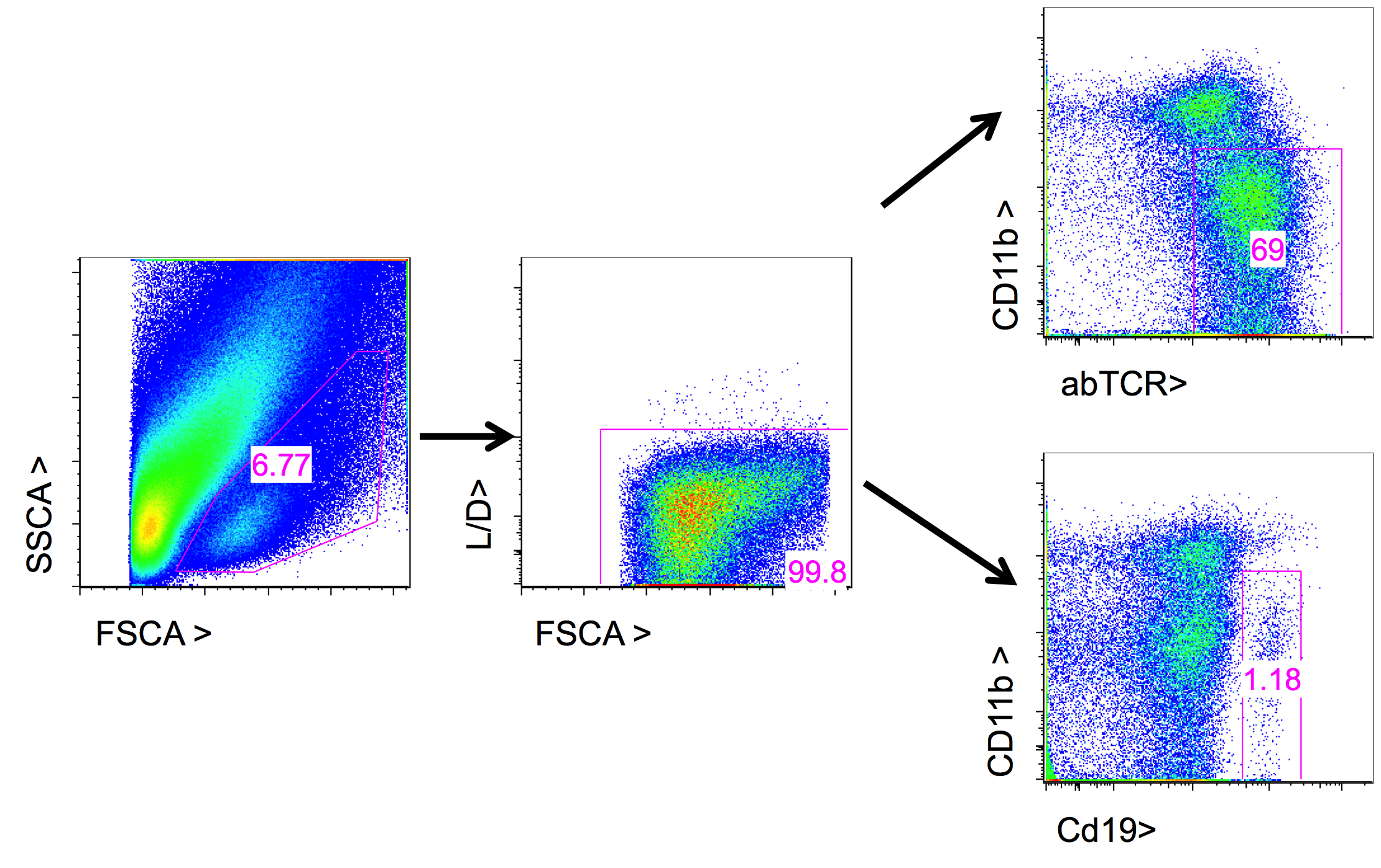

Supplement: S1 Fig — After kidney digestion and washing, cells were gated by scatter properties and live dead stain. CD11b+ myeloid cells, CD19+ B lymphocytes and TCR+ cells were analyzed as % of all leukocytes. T-cell receptor is expressed on both T-cell subtypes CD4 and CD8+ cells. Since by flow cytometric analysis both cell types are detected together the immunohistochemistry marker chosen was also CD3, which is expressed on all T-lymphocytes. (TIFF) [file pone.0162705.s001.tiff]

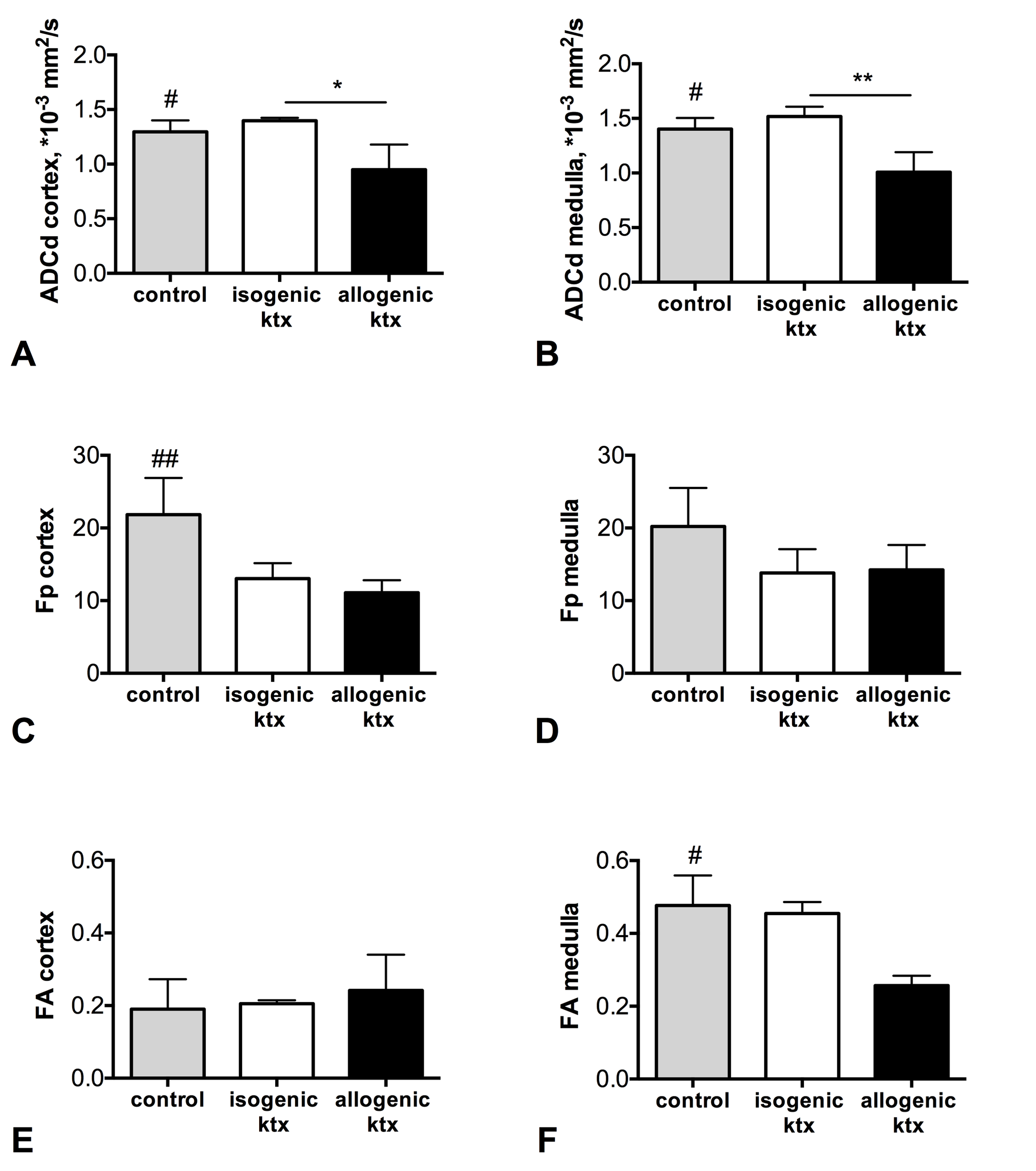

Supplement: S2 Fig — Depicted are mean ± SD of IVIM parameters ADCd (pure diffusion, A & B) and Fp (perfusion fraction, C & D) in renal cortex and renal medulla. In addition, FA is shown (fractional anisotropy, E & F). Significant differences are indicated *p<0.05, **p<0.01. Differences between control kidneys and allogenic kidney grafts are indicated with # p<0.05, ## p<0.01. (TIFF) [file pone.0162705.s002.tiff]
