# Supplementary material for: Multiparametric Functional MRI: Non-Invasive Imaging of Inflammation and Edema Formation after Kidney Transplantation in Mice
Source: PLoS One. 2016 Sep 15;11(9):e0162705. doi: 10.1371/journal.pone.0162705 (PMC5025122; doi:10.1371/journal.pone.0162705)
Supplement: S1 Text — The methods of IVIM and DTI are explained in Supporting File 1. (DOCX) [file pone.0162705.s004.docx]

**S1 File. IVIM analysis and DTI.**

**Methods:** In a subgroup of control, isogenic and allogenic ktx animals intravoxel incoherent motion (IVIM) analysis was performed to investigate the contribution of perfusion (perfusion fraction, Fp) and pure diffusion (ADCd) to ADC changes. We used a biexponential model with a multistep approach to calculate Fp, ADCd and ADCp (ADC perfusion) as described previously [1, 2]. In addition, in the same subgroup diffusion tensor imaging (DTI) was acquired using a respiratory-triggered, fat-saturated echo-planar sequence with the following parameters: field of view (FOV) = 40 x 35 mm^2^, slice thickness = 1.2 mm, number of averages = 4, matrix = 86 x 132, effective repetition time (TR)/ echo time (TE) = 2000-3000/ 33 ms, 2 b-values = 0 and 300 s/mm^2^. Parameter maps of the fractional anisotropy (FA) were calculated with MATLAB software (MathWorks, Natick, MA, USA) were calculated as described previously [3, 4]. IVIM parameters and FA were determined for renal cortex and outer medulla on an ROI based analysis using Osirix software (v.6.0.2, Pixmeo, Switzerland).

**References**

1. Hueper K, Khalifa AA, Brasen JH, Vo Chieu VD, Gutberlet M, Wintterle S, et al. Diffusion-Weighted imaging and diffusion tensor imaging detect delayed graft function and correlate with allograft fibrosis in patients early after kidney transplantation. J Magn Reson Imaging. 2016. doi: 10.1002/jmri.25158. PubMed PMID: 26778459.

2. Heusch P, Wittsack HJ, Pentang G, Buchbender C, Miese F, Schek J, et al. Biexponential analysis of diffusion-weighted imaging: comparison of three different calculation methods in transplanted kidneys. Acta Radiol. 2013;54(10):1210-7. Epub 2013/07/17. doi: 10.1177/0284185113491090. PubMed PMID: 23858509.

3. Notohamiprodjo M, Glaser C, Herrmann KA, Dietrich O, Attenberger UI, Reiser MF, et al. Diffusion tensor imaging of the kidney with parallel imaging: initial clinical experience. Investigative radiology. 2008;43(1536-0210; 1536-0210; 10):677-85.

4. Hueper K, Gutberlet M, Rodt T, Gwinner W, Lehner F, Wacker F, et al. Diffusion tensor imaging and tractography for assessment of renal allograft dysfunction-initial results. Eur Radiol. 2011;21(11):2427-33. Epub 2011/06/29. doi: 10.1007/s00330-011-2189-0. PubMed PMID: 21710264.
